# Public Support for Alcohol-Control Policies and Political Ideology in the US

**DOI:** 10.1001/jamahealthforum.2025.6436

**Published:** 2026-01-30

**Authors:** Joël Fokom Domgue, Robert Yu, Ernest Hawk, Sanjay Shete

**Affiliations:** 1 Department of Epidemiology, The University of Texas MD Anderson Cancer Center, Houston; 2 Division of Cancer Prevention and Population Sciences, The University of Texas MD Anderson Cancer Center, Houston; 3 Department of Biostatistics, The University of Texas MD Anderson Cancer Center, Houston; 4 Department of Clinical Cancer Prevention, The University of Texas MD Anderson Cancer Center, Houston

## Abstract

This cross-sectional study examines the role of US adults’ political affiliation, perception about alcohol use and cancer, and sociodemographic and behavioral factors in banning outdoor alcohol advertising and adding cancer warnings on alcohol containers.

## Introduction

Alcohol is a leading preventable cause of cancer in the US, responsible for about 100 000 cases and 25 000 deaths annually.^1^ Alcohol-related cancer burden has nearly doubled in the past few decades, consistent with increasing alcohol consumption, existing misbeliefs,^2^ and low public awareness of the association between alcohol use and cancer.^3^ Although alcohol-control policies could reduce the incidence and mortality of alcohol-related cancers, their implementation typically requires public support and legislative and executive action. To inform the adoption of 2 major alcohol-control policies, we examined public support for these policies and its association with political ideology, beliefs about the association between alcohol use and cancer, and other sociodemographic and behavioral factors.

## Methods

We analyzed data from the 2024 Health Information National Trends Survey (HINTS). Data were adjusted with sampling weights to improve representativeness. In accordance with the Common Rule (45 CFR §46), this cross-sectional study was exempt from ethics review and informed consent because we used deidentified, publicly available data. We followed the STROBE reporting guideline.

Support or opposition to 2 alcohol-control policies recommended in the 2025 US Surgeon General advisory^4^ was our main outcome, measured by asking: “To what extent would you support or oppose the following measures related to alcohol? Banning outdoor advertising of alcohol such as on billboards and bus stops, and requiring specific warnings about cancer on alcohol containers.” Main independent variables were political ideology (“Thinking about politics these days, how would you describe your own political viewpoint?”) and beliefs about alcohol’s association with cancer risk (“In your opinion, how does drinking alcohol affect the risk of getting cancer?”) Selected demographic and psychosocial characteristics were included as covariates.

Weighted proportions of public support or opposition to policies were estimated using SAS survey procedures (SAS Institute). Weighted multinomial logistic regression was used to examine the association of policy support or opposition with political ideology and beliefs about the association between alcohol use and cancer, adjusting for potential confounders. Two-tailed *P* < .05 indicated statistical significance. The eMethods in Supplement 1 provides additional details.

## Results

We included 7278 respondents (mean [SD] age, 49.0 [17.2] years; 2618 males [51.3%]). Among respondents, 2384 (33.9%) and 4250 (61.5%) supported, 3004 (45.7%) and 1874 (29.5%) neither supported nor opposed, and 1330 (20.4%) and 584 (8.9%) opposed banning outdoor alcohol advertising and adding cancer-specific warnings on alcoholic containers, respectively.

Proportions of individuals who supported, neither supported nor opposed, or opposed these alcohol-control policies varied according to their political ideology and beliefs about the association between alcohol use and cancer risk (Figure). Compared with liberal respondents, conservative respondents were more likely to oppose banning outdoor advertising (adjusted odds ratio [AOR], 1.88; 95% CI, 1.27-2.79; *P* = .002) and adding cancer-specific warning labels (AOR, 1.87; 95% CI, 1.20-2.90; *P* = .006). Respondents who believed that alcohol had “no effect on cancer risk” had higher odds of opposition to banning outdoor advertising (AOR, 3.19; 95% CI, 2.01-5.07; *P* < .001) and adding cancer-specific warning labels (AOR, 3.50; 95% CI, 1.96-6.24; *P* < .001). Alcohol consumption, cigarette smoking, and being male were also associated with opposition to these alcohol-control policies (Table).

**Figure.**
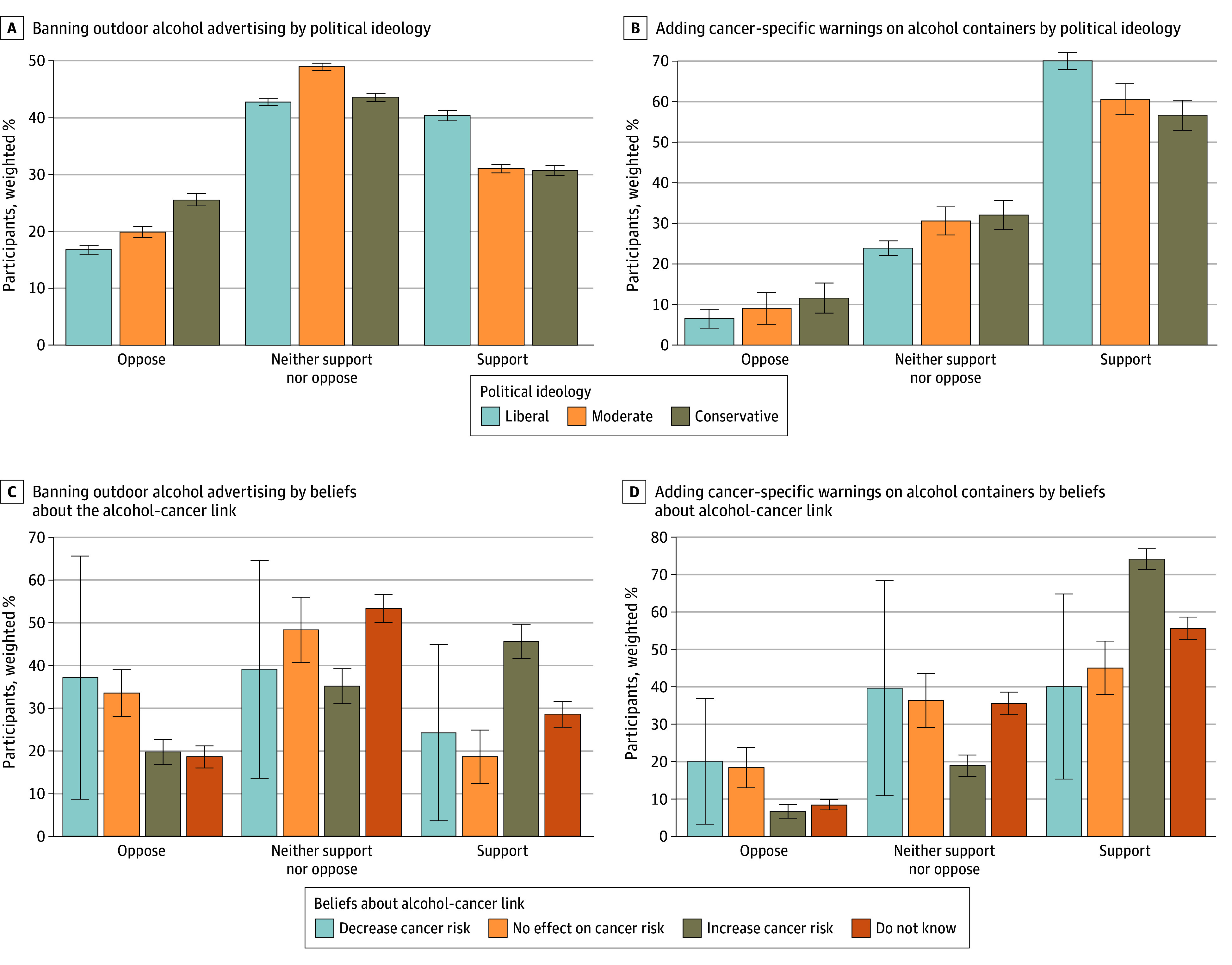
Support or Opposition to Alcohol-Control Policies According to Political Ideology and Beliefs About Alcohol and Cancer Link in the US Adult Population Error bars represent 95% CIs.

**Table.** Multinomial Logistic Regression Analysis of Factors in Support for or Opposition to Alcohol-Control Policies Among US Adults in the 2024 HINTS

Variable	Banning outdoor alcohol advertising				Adding cancer-specific warnings on alcohol containers			
	Oppose^a^		Neutral		Oppose^a^		Neutral	
	OR (95% Cl)	*P* value	OR (95% Cl)	*P* value	OR (95% Cl)	*P* value	OR (95% Cl)	*P* value
Political ideology								
Conservative	1.88 (1.27-2.79)	.002	1.23 (0.94-1.60)	.13	1.87 (1.20-2.90)	.006	1.33 (1.02-1.74)	.04
Liberal	1 [Reference]		1 [Reference]		1 [Reference]		1 [Reference]	
Moderate	1.50 (0.96-2.33)	.07	1.29 (0.96-1.71)	.09	1.41 (0.81-2.45)	.22	1.25 (0.95-1.64)	.11
“In your opinion, how much does drinking affect the risk of getting cancer?”								
Increase cancer risk	1 [Reference]		1 [Reference]		1 [Reference]		1 [Reference]	
Decrease cancer risk	2.82 (0.69-11.47)	.14	2.05 (0.47-8.90)	.33	3.19 (0.86-11.87)	.08	3.02 (0.85-10.72)	.09
No effect on cancer risk	3.19 (2.01-5.07)	<.001	2.97 (1.71-5.16)	<.001	3.50 (1.96-6.24)	<.001	2.66 (1.83-3.86)	<.001
Do not know	1.51 (1.12-2.03)	.008	2.35 (1.78-3.11)	<.001	1.52 (1.00-2.30)	.05	2.30 (1.75-3.01)	<.001
Past-month alcohol drinking^b^								
Yes	2.75 (2.12-3.57)	<.001	1.76 (1.44-2.14)	<.001	1.49 (1.04-2.16)	.03	1.48 (1.16-1.89)	.002
No	1 [Reference]		1 [Reference]		1 [Reference]		1 [Reference]	
Age, y								
≤34	1.57 (1.01-2.42)	.04	1.41 (1.02-1.96)	.04	1.20 (0.68-2.10)	.52	0.96 (0.68-1.35)	.81
35-49	0.98 (0.69-1.39)	.90	1.27 (0.91-1.78)	.16	0.93 (0.54-1.60)	.78	1.10 (0.83-1.47)	.50
50-64	1.10 (0.78-1.54)	.59	1.19 (0.93-1.53)	.17	0.72 (0.45-1.18)	.19	1.04 (0.80-1.35)	.77
≥65	1 [Reference]		1 [Reference]		1 [Reference]		1 [Reference]	
Sex								
Male	1.62 (1.30-2.02)	<.001	0.97 (0.77-1.21)	.75	1.73 (1.25-2.38)	.001	1.04 (0.82-1.32)	.76
Female	1 [Reference]		1 [Reference]		1 [Reference]		1 [Reference]	
Race and ethnicity^c^								
Hispanic	0.74 (0.52-1.05)	.09	0.93 (0.68-1.26)	.62	0.89 (0.52-1.51)	.65	0.77 (0.56-1.05)	.10
Non-Hispanic Asian	0.70 (0.25-1.96)	.48	0.94 (0.55-1.61)	.82	1.44 (0.32-6.53)	.63	1.24 (0.69-2.24)	.47
Non-Hispanic Black	1.11 (0.70-1.74)	.66	1.21 (0.86-1.70)	.27	0.98 (0.48-2.04)	.97	0.65 (0.47-0.89)	.008
Non-Hispanic White	1 [Reference]		1 [Reference]		1 [Reference]		1 [Reference]	
Non-Hispanic Other^d^	1.31 (0.73-2.34)	.35	1.25 (0.76-2.06)	.37	1.08 (0.49-2.42)	.84	0.96 (0.55-1.67)	.88
Educational level								
Postgraduate degree	1 [Reference]		1 [Reference]		1 [Reference]		1 [Reference]	
High school diploma and post high school	1.15 (0.80-1.65)	.46	1.41 (1.05-1.89)	.03	1.20 (0.71-2.03)	.49	1.23 (0.94-1.62)	.13
College degree	1.19 (0.82-1.75)	.36	1.03 (0.77-1.37)	.86	1.50 (0.97-2.34)	.07	1.15 (0.88-1.48)	.30
Household income, $								
<35 000	0.71 (0.46-1.11)	.13	0.68 (0.51-0.90)	.007	1.27 (0.78-2.08)	.32	0.85 (0.64-1.14)	.28
35 000 to <50 000	1.04 (0.72-1.51)	.83	0.98 (0.69-1.39)	.92	1.12 (0.62-2.01)	.71	0.96 (0.65-1.41)	.83
50 000 to <75 000	0.77 (0.55-1.10)	.15	0.85 (0.67-1.09)	.19	0.86 (0.48-1.54)	.60	0.83 (0.60-1.15)	.26
≥75 000	1 [Reference]		1 [Reference]		1 [Reference]		1 [Reference]	
Residence								
Rural	1.07 (0.80-1.44)	.63	1.14 (0.89-1.46)	.30	1.22 (0.80-1.85)	.34	1.58 (1.30-1.91)	<.001
Urban	1 [Reference]		1 [Reference]		1 [Reference]		1 [Reference]	
Cigarette smoking^e^								
Current smoker	1.45 (0.91-2.30)	.12	1.51 (1.06-2.16)	.02	1.92 (1.17-3.15)	.01	1.62 (1.08-2.43)	.02
Former smoker	1.52 (1.09-2.13)	.01	1.20 (0.91-1.59)	.20	1.70 (1.20-2.40)	.003	1.34 (1.00-1.80)	.047
Never smoker	1 [Reference]		1 [Reference]		1 [Reference]		1 [Reference]	
“It seems like everything causes cancer.”								
Strongly/somewhat disagree	1.04 (0.77-1.39)	.82	0.90 (0.71-1.14)	.36	0.96 (0.65-1.41)	.83	0.93 (0.71-1.22)	.60
Strongly/somewhat agree	1 [Reference]		1 [Reference]		1 [Reference]		1 [Reference]	
“There’s not much you can do to lower your chances of getting cancer.”								
Strongly/somewhat agree	1.39 (1.03-1.88)	.03	1.00 (0.77-1.30)	.99	2.07 (1.30-3.30)	.003	1.71 (1.31-2.21)	<.001
Strongly/somewhat agree	1 [Reference]		1 [Reference]		1 [Reference]		1 [Reference]	
“There are so many different recommendations about preventing cancer, it’s hard to know which ones to follow.”								
Strongly/somewhat disagree	0.98 (0.72-1.32)	.88	0.84 (0.65-1.09)	.18	1.08 (0.74-1.57)	.68	1.19 (0.89-1.58)	.23
Strongly/somewhat agree	1 [Reference]		1 [Reference]		1 [Reference]		1 [Reference]	
“When I think about cancer, I automatically think about death.”								
Strongly/somewhat disagree	1.10 (0.86-1.41)	.44	1.10 (0.88-1.38)	.39	1.47 (1.05-2.07)	.03	1.29 (1.01-1.66)	.04
Strongly/somewhat agree	1 [Reference]		1 [Reference]		1 [Reference]		1 [Reference]	
“In the past 12 months, not counting times you went to an emergency department, how many times did you see a doctor, nurse, or other health professional to get care for yourself?”								
None	1 [Reference]		1 [Reference]		1 [Reference]		1 [Reference]	
1-2	0.95 (0.54-1.68)	.86	1.10 (0.72-1.68)	.64	0.95 (0.53-1.72)	.87	0.84 (0.56-1.26)	.39
≥3	0.74 (0.44-1.24)	.25	1.03 (0.68-1.57)	.89	0.88 (0.51-1.52)	.64	0.87 (0.57-1.32)	.50
“Have you ever been diagnosed as having cancer?”								
Yes	0.97 (0.66-1.42)	.85	0.89 (0.65-1.22)	.46	0.77 (0.51-1.16)	.21	1.07 (0.76-1.53)	.68
No	1 [Reference]		1 [Reference]		1 [Reference]		1 [Reference]	
“Have any of your first- or second-degree biological relatives ever had cancer?”								
No	1.14 (0.79-1.64)	.48	1.02 (0.80-1.30)	.89	1.38 (0.88-2.15)	.16	1.19 (0.90-1.58)	.22
Not sure	0.80 (0.51-1.25)	.32	1.25 (0.88-1.79)	.21	1.00 (0.51-1.96)	.99	1.75 (1.21-2.53)	.004
Yes	1 [Reference]		1 [Reference]		1 [Reference]		1 [Reference]	

Abbreviations: HINTS, Health Information National Trends Survey; OR, odds ratio.

^a^ The reference category for the outcome variables is support for alcohol-control policies.

^b^ Past-month alcohol drinking was measured using 2 items: (1) “During the past 30 days, how many days per week did you have at least one drink of any alcoholic beverage?” and (2) “During the past 30 days, on the days when you drank, about how many drinks did you drink on average?” Respondents were categorized as past-month drinkers (at least 1 drink at least 1 day per week in the past 30 days), and past-month nondrinkers (no alcohol drink in the past 30 days).

^c^ Race and ethnicity data were self-identified by HINTS respondents as follows: 1323 (17.5%) Hispanic, 342 (5.6%) non-Hispanic Asian, 968 (11.1%) non-Hispanic Black, 3548 (60.6%) non-Hispanic White, and 257 (53.3%) non-Hispanic other. This variable was included in this study because it has been reported to play a role in the support or opposition for public health policies.

^d^ Non-Hispanic other includes American Indian or Alaska Native, Native Hawaiian, Guamanian or Chamorro, Samoan, Other Pacific Islander, as reported by respondents.

^e^ Respondents who reported having smoked fewer than 100 cigarettes in their entire life and who were not smoking at the time of the survey were classified as never smokers. Those who reported having smoked at least 100 cigarettes in their entire life and who were not smoking at the time of the survey were classified as former smokers. Those who reported smoking cigarettes every day or some days at the time of the survey were classified as current smokers.

## Discussion

Among US adults, public support was low for alcohol-control policies, particularly banning outdoor alcohol advertising. Additionally, support or opposition to these policies varied according to political ideology, beliefs about the association between alcohol use and cancer, and alcohol consumption.

These findings call for tailored communication strategies, involving patient and clinician discussions and mass-media campaigns, to raise public awareness about alcohol’s association with cancer and boost political support for regulatory actions regarding alcohol consumption, possibly mitigating oppositional influences from commercial and other interests.^5^ Moreover, our findings suggest that bipartisan buy-in is necessary to enact these policies. Since their adoption requires political endorsement,^6^ mobilizing national opinion on alcohol’s association with cancer is needed to accelerate implementation of policies endorsed by the Surgeon General.

A study limitation was the cross-sectional design of HINTS. It prevented ascertainment of a causal association between support for alcohol-control policies and independent variables.

## Notes

Supplement 1.
eMethods

eReferences

Supplement 2.
Data Sharing Statement
