# Supplementary material for: Public Support for Alcohol-Control Policies and Political Ideology in the US
Source: JAMA Health Forum. 2026 Jan 30;7(1):e256436. doi: 10.1001/jamahealthforum.2025.6436 (PMC12859717; doi:10.1001/jamahealthforum.2025.6436)
Supplement: Supplement 1. — eMethods eReferences [file jamahealthforum-e256436-s001.pdf]

Supplemental Online Content

Fokom Domgue J, Yu R, Hawk E, Shete S. Public support for alcohol-control policies and political ideology in the US. *JAMA Health Forum*. 2026;7(1):e256436. doi:10.1001/jamahealthforum.2025.6436

eMethods

eReferences

This supplementary material has been provided by the authors to give readers additional details about their work.

© 2026 Fokom Domgue J et al. *JAMA Health Forum*

eMethods

Data source and Study population

This survey study was based on the 2024 Health Information National Trends Survey (HINTS) data, a nationally representative household survey of US adults in the civilian non-institutionalized population conducted by the US National Cancer Institute (Data released in May 2025). The HINTS 7 employed a two-stage sampling design with a stratified sample of addresses selected in the first stage and one adult selected within each sampled household.[1] The household addresses were divided into four sampling strata: (1) addresses in urban areas with a high concentration of minorities, (2) addresses in urban areas with a low concentration of minorities, (3) addresses in rural areas with a high concentration of minorities, and (4) addresses in rural areas with a low concentration of minorities. Sampling weights adjusting for non-response and non-coverage biases were employed to improve representativeness.[1] Respondents completed the survey online or on paper, and the data were collected between March and September 2024. Since these data are deidentified and publicly available, our study did not require institutional review board review.[2] We included all individuals aged 18 years and older who responded to questions assessing their support or opposition to two alcohol reduction policies endorsed by the US Surgeon General in his January 2025 advisory,[3] namely "banning outdoor alcohol advertising" and "requiring cancer-specific warnings on alcohol containers". Of the 7278 individuals who took the HINTS 7 survey, 560 and 570 individuals (less than 7% of the total respondents) did not respond to questions about their support/neutrality/opposition to

© 2026 Fokom Domgue J et al. *JAMA Health Forum*

banning outdoor alcohol advertising and their support/neutrality/opposition to requiring cancer-specific warning labels on alcoholic beverages, respectively, and were excluded from our study sample. Our study followed the STROBE reporting guidelines.[4]

Measures

Outcome variables

Support for or opposition to two alcohol control policies (banning outdoor alcohol advertising, and adding cancer-specific warning labels on alcohol containers) were our outcome variables. They were measured with the question: *"To what extent would you support or oppose the following measures (A. Banning outdoor advertising of alcohol such as on billboards and bus stops? B. Requiring specific warnings about cancer on alcohol containers) related to alcohol?"* Response options to this question were: "strongly oppose", "oppose", "neither support nor oppose", "support", and "strongly support". In this analysis, responses were recoded into three categories: oppose (oppose/strongly oppose), neutral (neither support nor oppose), and support (support/strongly support).

Primary predictors

The two predictor variables for this study were political ideology and beliefs about the effect of alcohol on cancer risk. Political ideology was measured with the question: *"Thinking about politics these days, how would you describe your own political viewpoint?"* Possible responses to this question (very liberal, liberal, somewhat liberal, moderate, somewhat conservative/ conservative/very conservative) were recoded into three

© 2026 Fokom Domgue J et al. *JAMA Health Forum*

categories: liberal (very liberal/liberal/somewhat liberal), moderate (moderate), and conservative (somewhat conservative/ conservative/very conservative). Beliefs about the effect of alcohol on cancer risk was measured by asking: *"In your opinion, how does drinking alcohol affect the risk of getting cancer?"* Possible responses to this question were: "decreases risk of cancer", "has no effect on cancer risk", "increases risk of cancer", and "don't know". The variable "Beliefs about the effect of alcohol on cancer risk" was also considered a primary predictor because it has proven to have implications for alcohol control policy support, adoption and implementation in other high-income settings.[5,6]

Covariates

Selected sociodemographic, behavioral, and health-related variables were used to describe the study population according to their opposition or support for alcohol control policies. Specifically, age in years (<35; 35 – 49; 50 – 64; ≥ 65), sex (Male; Female), race/ethnicity (non-Hispanic Whites; non-Hispanic Blacks; Hispanics; non-Hispanic Asians; and non-Hispanic Others which includes the following racial groups: American Indian or Alaska Native, Native Hawaiian, Guamanian or Chamorro, Samoan, Other Pacific Islander, who reported their ethnicity as non-Hispanic), level of education (up to high school/post-high school or some college; college graduate; postgraduate), place of residence (urban, rural); annual household income (<$35,000; $35,000 - < $50,000; $50,000 - < $75,000 ; ≥ $75,000), personal cancer history (no; yes), family cancer history (yes; no; don't know), were used to describe the study population.

© 2026 Fokom Domgue J et al. *JAMA Health Forum*

Given the high rates of alcohol consumption among cigarette smokers, this study also adjusted for alcohol consumption and cigarette smoking. Alcohol consumption was measured as past-month alcohol drinking, using two questions: (1) "*During the past 30 days, how many days per week did you have at least one drink of any alcoholic beverage?"*; and (2) *"During the past 30 days, on the days when you drank, about how many drinks did you drink on average?"* For the variable "alcohol consumption", respondents were categorized as past-month drinkers (consuming at least one alcoholic drink at least one day per week in the past 30 days), and past-month non-drinkers (no alcohol drink in the past 30 days).

Concerning the variable "cigarette smoking", those who reported having smoked fewer than 100 cigarettes in their entire life and who were not smoking at the time of survey, were classified as never smokers; those who reported having smoked at least 100 cigarettes in their entire life and who were not smoking at the time of survey, were classified as former smokers; while those who reported smoking cigarettes every day or some days at the time of survey, were classified as current smokers.

Interaction with the healthcare system, measured by the frequency of non-emergency visits to a healthcare professional in the past 12 months (none; one or two; three or more), was also included as a covariate. In addition, individuals' beliefs about cancer were measured using the following questions/statements: It seems like everything causes cancer (Strongly/Somewhat agree; Strongly/Somewhat disagree), there's not much you can do to lower your chances of getting cancer (Strongly/Somewhat agree; Strongly/Somewhat disagree), there are so many different recommendations about

© 2026 Fokom Domgue J et al. *JAMA Health Forum*

preventing cancer, it's hard to know which ones to follow (Strongly/Somewhat agree; Strongly/Somewhat disagree), and when I think about cancer, I automatically think about death (Strongly/Somewhat agree; Strongly/Somewhat disagree). These variables were considered covariates based on previously reported associations with cancer risk beliefs and perceptions.[7,8]

Missing data

For the outcome variables, individuals with missing data were excluded from the study sample. For each predictor variable and covariate included in our analyses, a "missing" category was created during regression analyses, thereby keeping them in the analysis. This strategy allows us to treat "missingness" as an informative category in the model, especially when the missingness might be meaningful or potentially correlated with the outcome variable.

Statistical analysis

Weighted descriptive statistics were used to characterize the study population according to their support or opposition to these two alcohol control policies. Variations in the prevalence of public support for these two policies were estimated according to political ideology, alcohol consumption, and beliefs about the effect of alcohol use on cancer risk. A survey-weighted multinomial logistic regression analysis using survey procedures in SAS

© 2026 Fokom Domgue J et al. *JAMA Health Forum*

software (SAS 9.4) was performed to examine the association of public support for alcohol control policies with political ideology and beliefs about the alcohol-cancer link, with the above-mentioned covariates included in the model. In this analysis, the outcome variables were classified into three categories (support, neutral, and oppose), with "support" being the reference category. Adjusted odds ratios (aORs) and 95% confidence intervals (CIs) were computed, with the corresponding p-values. Statistical tests were two-tailed, and a $P<.05$ was considered statistically significant. Data were analyzed between July 25, 2025, and August 14, 2025.

© 2026 Fokom Domgue J et al. *JAMA Health Forum*

eReferences

1. Health Information National Trends Survey 7 (HINTS 7) Methodology Report. Available at: https://hints.cancer.gov/docs/methodologyreports/HINTS_7_MethodologyReport.pdf. Lastly accessed on August 15, 2025.
2. US Department of Health and Human Services Office for Human Research Protections. Human subject regulations. 2022. Available at: https://www.hhs.gov/ohrp/regulations-and-policy/index.html. Last accessed on March 3, 2024.
3. January 3, 2025. US Department of Health and Human Services. New Surgeon General's Advisory on Alcohol and Cancer Risk. Available at: https://www.hhs.gov/about/news/2025/01/03/us-surgeon-general-issues-new-advisory-link-alcohol-cancer-risk.html. Last accessed on July 12, 2025.
4. Ghaferi AA, Schwartz TA, Pawlik TM. STROBE Reporting Guidelines for Observational Studies. *JAMA Surg.* 2021;156(6):577-578.
5. Bates S, Holmes J, Gavens L, et al. Awareness of alcohol as a risk factor for cancer is associated with public support for alcohol policies. *BMC Public Health.* 2018;18(1):688.
6. Buykx P, Li J, Gavens L, et al. Public awareness of the link between alcohol and cancer in England in 2015: a population-based survey. *BMC Public Health.* 2016;16(1):1194.
7. Wiseman KP, Klein WMP. Evaluating Correlates of Awareness of the Association between Drinking Too Much Alcohol and Cancer Risk in the United States. *Cancer Epidemiol Biomarkers Prev.* 2019;28(7):1195-1201.

© 2026 Fokom Domgue J et al. *JAMA Health Forum*

8. Kiviniemi MT, Orom H, Hay JL, Waters EA. Limitations in American adults' awareness of and beliefs about alcohol as a risk factor for cancer. *Prev Med Rep.* 2021;23:101433.

© 2026 Fokom Domgue J et al. *JAMA Health Forum*
