# Supplementary material for: Public Support for Alcohol-Control Policies and Political Ideology in the US
Source: JAMA Health Forum. 2026 Jan 30;7(1):e256436. doi: 10.1001/jamahealthforum.2025.6436 (PMC12859717; doi:10.1001/jamahealthforum.2025.6436)
Supplement: Supplement 2. — Data Sharing Statement [file jamahealthforum-e256436-s002.pdf]

Data Sharing Statement

Fokom Domgue. Public Support for Alcohol-Control Policies and Political Ideology in the US. *JAMA Health Forum*. Published January 30, 2026. doi:10.1001/jamahealthforum.2025.6436

Data
Data available: No
